# Neurorehabilitation in Parkinson's Disease: A Critical Review of Cognitive Rehabilitation Effects on Cognition and Brain

**DOI:** 10.1155/2018/2651918

**Published:** 2018-05-06

**Authors:** María Díez-Cirarda, Naroa Ibarretxe-Bilbao, Javier Peña, Natalia Ojeda

**Affiliations:** Department of Methods and Experimental Psychology, Faculty of Psychology and Education, University of Deusto, Bilbao, Spain

## Abstract

**Background:**

Parkinson's disease (PD) patients experience cognitive impairment which has been related to reduced quality of life and functional disability. These symptoms usually progress until dementia occurs. Some studies have been published assessing the efficacy of cognitive treatments on improving cognition, functional outcome, and producing changes in brain activity.

**Objective:**

A critical review was performed to present up-to-date neurorehabilitation effects of cognitive rehabilitation in PD, with special emphasis on the efficacy on cognition, quality of life aspects, brain changes, and the longitudinal maintenance of these changes.

**Results:**

After exclusions, 13 studies were reviewed, including 6 randomized controlled trials for the efficacy on cognition, 2 randomized controlled trials regarding the brain changes after cognitive training, and 5 studies which evaluated the long-term effects of cognitive treatments.

**Conclusions:**

Cognitive rehabilitation programs have demonstrated to be effective on improving cognitive functions, but more research is needed focusing on the efficacy on improving behavioral aspects and producing brain changes in patients with PD. Moreover, there is a need of randomized controlled trials with long-term follow-up periods.

## 1. Introduction

Parkinson's disease (PD) is a common neurodegenerative disease, being most of the cases diagnosed at around 60 years [1]. Traditionally, PD has been considered a motor disorder, and the core motor symptoms are rigidity, tremor, bradykinesia (akinesia), and postural instability. In addition, freezing of gait (difficulty to initiate or continue walking) and flexed posture have been included in the cardinal motor symptoms of the disease [2]. Nowadays, it is known that this neurodegenerative process produces a wide range of motor and nonmotor symptoms in PD patients; hence, PD is considered a multiple system neurodegenerative disorder [3]. Among these nonmotor symptoms, cognitive impairment is an important nonmotor symptom due to its prevalence among PD patients (20–50%) [4, 5]. In addition, PD patients might develop cognitive impairment from the early stages of the disease [4, 5]. These cognitive deficits may deteriorate with the progression of the disease until dementia occurs [6, 7]. The analysis of cognitive impairment and dementia in PD patients is relevant because both have shown relationship with reduced quality of life and functional disability in PD patients [8, 9].

These cognitive impairments in PD have been related to grey matter (GM) atrophy, white matter (WM) alterations, brain functional connectivity (FC), and brain activation alterations. PD patients with Mild Cognitive Impairment (MCI) diagnosis have shown GM volume reduction in the frontal, temporal, and parietal lobes, but also in the hippocampus, amygdala, and putamen [10, 11]. Additionally, PD patients with MCI have shown widespread cerebral WM deterioration [11–13]. Interestingly, WM alterations have been found to appear before GM volume reduction in PD patients, which highlights the importance to explore the relationship between WM indexes and cognitive impairment [14]. In addition, cognitive deficits have also been related to functional brain alterations, showing altered FC and brain activation values both during resting state and during cognitive tasks inside the scanner [15, 16].

With the progression of the disease, cognitive deficits usually deteriorate until dementia occurs after 10 to 20 years [6, 17]. A study followed newly diagnosed PD patients over time and found that after 20 years, dementia was present in up to 80% of PD patients [6]. In addition, recent studies showed that the presence of MCI diagnosis in PD patients contributes to the development of dementia [7], and results support that MCI could be considered as a prodromal stage for dementia in PD [18]. Cognitive deterioration is accompanied by GM volume loss [19], WM alterations [20], and functional brain changes [21, 22]. When dementia occurs in PD patients, cortical degeneration has been extended to frontal, temporal, parietal, and occipital areas [23].

Due to the relevance of cognitive deficits, therapeutic strategies are needed to treat cognitive decline. A common cognitive rehabilitation program could be described as a behavioral treatment for cognitive impairment which focused on cognitive abilities and daily living activities, which is based on the restoration, compensation, and optimization of the cognitive functions [24, 25]. Cognitive rehabilitation programs have demonstrated their efficacy on improving cognition in different studies in PD. Several reviews [26–28] and meta-analyses [29, 30] have been published in the field. The efficacy of cognitive rehabilitation on improving cognition has been shown, but these reviews and meta-analyses highlight the importance of continuing with research focused on the efficacy of the cognitive rehabilitation approach in PD.

The main objective of the present study is to perform a critical review to present up-to-date neurorehabilitation effects of cognitive rehabilitation in PD. The first objective was to examine the efficacy of cognitive rehabilitation programs on cognition and behavioral aspects. The second objective was to review the evidence of the brain changes found after cognitive treatments. Finally, the present study analyzed the long-term effects of cognitive rehabilitation in PD.

## 2. Methods

### 2.1. Review Strategy

Studies were included from inception to December 2017. Focusing on the first objective of this critical review, which was to analyze those randomized controlled trials focused on the efficacy of cognitive rehabilitation programs on cognition, we selected only those studies that fulfilled the following criteria: (1) randomized controlled trials; (2) PD patients underwent a cognitive rehabilitation program; (3) the main objective was to investigate the change in cognition; and (4) studies including a PD control group. Among the studies that fulfilled these specific criteria, we also reported (if included in the studies) the results of the efficacy on behavioral or mood aspects, such as depression, apathy, functional disability, and quality of life aspects.

Regarding the second objective of the present review, the efficacy of cognitive rehabilitation programs in producing brain changes in PD was determined based on the following criteria: (1) randomized controlled trials; (2) PD patients underwent a cognitive rehabilitation program; (3) studies including a PD control group; and (4) brain changes were evaluated.

Finally, focusing on the last objective of the present study, the review about the long-term effects of cognitive rehabilitation programs was based on the following criteria: (1) PD patients underwent a cognitive rehabilitation program; (2) a longitudinal follow-up evaluation was performed; and (3) the main objective was to investigate the change in cognition at follow-up. In this specific section, we included both randomized controlled trials and nonrandomized trials, due to the scarce number of published studies and to have a wider perspective.

Databases included were PubMed, Medline, and Google Scholar. The search terms were specified to be found in the title of the studies. The terms were (1) Parkinson's disease/Parkinson disease; (2) cognitive rehabilitation/cognitive training/cognitive remediation; (3) attention rehabilitation/attention training/attention remediation; (4) executive training/executive rehabilitation/executive remediation; (5) memory training/memory rehabilitation/memory remediation; (6) randomized controlled trial/randomized controlled trial; and (7) cognition. The search term combinations in the databases were (1) + (2); (1) + (3); (1) + (4); (1) + (5); and (1) + (6) + (7).

**Figure 1:**
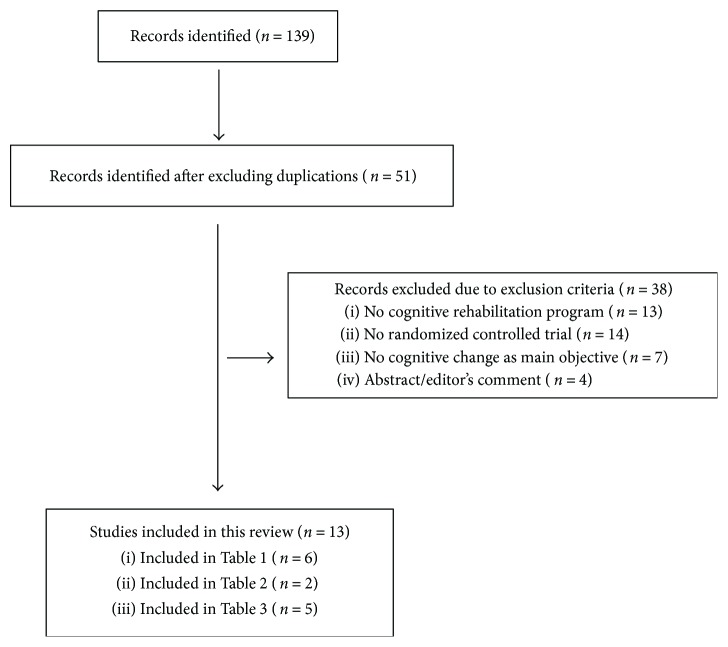
Summary of studies identified and included in the review.

A summary of study selection is shown in Figure 1. The results of the selected studies were divided into 3 different sections. First, the studies evaluating the cognitive and behavioral changes are shown in Table 1. Then, Table 2 shows the studies that assessed the brain changes after cognitive rehabilitation in PD. In addition, the longitudinal effects of cognitive rehabilitation programs are shown in Table 3. In Tables 1 and 3, different characteristics of the studies are shown, such as the sample size, characteristics of the cognitive rehabilitation program used, cognitive domains analyzed, significant results found, and limitations of each study. In Table 2, MRI acquisition, preprocessing, and analysis specifications are included for each study, along with the brain significant results and the study limitations.

## 3. Results

### 3.1. Efficacy on Improving Cognition and Behavioral Aspects

A summary of the included cognitive rehabilitation studies in PD is shown in Table 1. Studies were included if they followed guidelines for randomized controlled trials, the intervention was a cognitive rehabilitation program, and the main objective of the study was to improve cognition. As previously reported in other reviews and meta-analyses, cognitive rehabilitation improves cognition in PD [27, 29, 30]. However, there is a need for studies with larger samples and double-blind randomized controlled trials to reach generalized conclusions in PD.

A less studied aspect of cognitive rehabilitation is its efficacy on improving mood symptoms or functional disability related to the disease. Following the review-specific criteria, among the randomized controlled trials in PD, only five studies have evaluated the change in functionality and mood aspects [31–35] and two of them found positive effects [31, 32] (Table 1). Petrelli et al. compared a structured and a nonstructured cognitive training program in PD patients and found that the symptoms of depression were reduced only in those PD patients that attended the nonstructured cognitive program [32]. Peña et al. found that functional disability scores were reduced in the experimental group (3 months of cognitive training) compared to the active control group [31]. On the contrary, París et al. evaluated the change in quality of life, depressive symptoms, and activities of daily life after attending a cognitive rehabilitation program [35]. No significant changes were found in any scale, and authors related the absence of significant changes in quality of life to the short time of training (12 sessions in 1 month). In the same line, PD patients in the study of Cerasa et al. also attended a cognitive training program during 12 sessions and showed no significant changes in mood status [33]. However, in the study of Edwards et al., PD patients attended a cognitive training program during a longer period of time (3 months), but patients showed no changes in behavioral measures [34]. Among clinical symptoms of the disease, the change in depressive symptoms has been usually assessed in cognitive training studies in PD, but despite some significant changes, the overall results point to the absence of efficacy in reducing depression symptomatology after treatment [30]. However, these studies excluded patients with depression diagnosis or with severe symptoms of depression prior to participation. Therefore, this criterion could have influenced the absence of significant changes. With all, the mechanisms that make possible the improvement in quality of life aspects after a cognitive rehabilitation program are not clear. The duration of treatment and degree of structuration of the sessions could be two relevant variables to take into account when assessing transfer effects to clinical variables, but other variables seem to influence this process. Interestingly, in schizophrenia studies, the presence of a therapist during the training sessions and the group format of the training program have been suggested to influence the results on mood symptoms [36]. The cognitive sessions carried in a group format enhance social interactions between participants, and the presence of a therapist may increase the motivation and give positive feedback to the patients, which could have an impact in the affective state of patients. In fact, the two PD studies that found transfer effects to functional aspects or depressive symptoms performed a group-based cognitive training, and the training was guided by a qualified therapist [31, 32].

Moreover, detecting variables that predict the efficacy of cognitive treatments is an important aspect to take into account in order to understand the cognitive rehabilitation process, which could guide researchers to develop more effective programs and clinicians to personalize treatments for patients (Table 1). Despite the large amount of studies assessing the efficacy of cognitive rehabilitation in PD, few studies have investigated the predictors of the efficacy of cognitive treatments in PD. These PD studies found that lower age at diagnosis and longer disease duration were predictors of higher degree of cognitive improvements after rehabilitation [34], but higher scores in working memory and flexibility at baseline were related with lower degree of improvements after rehabilitation [37].

### 3.2. Changes in Brain Activity after Cognitive Rehabilitation

Little is known about the neurobiological effects of cognitive rehabilitation programs in PD. To date, literature is scarce about the presence of cerebral changes associated with cognitive rehabilitation programs assessed with structural and functional MRI techniques in PD. Table 2 summarized the main findings of the two randomized controlled trials in evaluating brain changes after a cognitive rehabilitation program in PD.

One study evaluated the effects of group-based attention rehabilitation on brain functional activity in PD patients [33]. PD patients were included in the trial if they had attention impairment but no other cognitive domain impaired. At pre- and post-treatment assessments, patients underwent an extensive neuropsychological assessment and resting-state fMRI were acquired. PD patients were randomly divided into experimental group and active control group. The experimental group received attention rehabilitation using “RehaCom” computer program, while the control group attended in-house software which focused on visuomotor coordination. The attention rehabilitation consisted in computer-assisted tasks which trained attention and information processing during 6 weeks. Specifically, attention rehabilitation tasks were focused on concentration and attention tasks and vigilance program and divided attention from the RehaCom software. After rehabilitation, PD patients showed improvements in attention and processing speed tasks and increased brain activation in the left dorsolateral prefrontal cortex (part of the executive resting-state network) and the left superior parietal cortex (part of the attentional resting-state network) [33] (Table 2).

A later study in PD patients evaluated the changes in brain activity after a 3-month integrative cognitive rehabilitation program [38]. PD patients underwent an extensive neuropsychological assessment at pre- and post-treatment. Regarding MRI acquisition, GM and WM changes were analyzed as well as brain activity changes during resting-state and during a memory paradigm. The cognitive rehabilitation program used was the REHACOP, a paper/pencil rehabilitation program, which trained attention, processing speed, memory, language, executive functions, and social cognition during 3 months. PD patients after cognitive rehabilitation showed increased brain FC between frontal and temporal lobes and increased brain activation during the memory paradigm in frontal and temporal areas (see Table 2). No brain structural changes were found after rehabilitation. These brain FC and activation values at post-treatment showed correlations with post-treatment cognitive performance in PD patients from the experimental group. Specifically, during resting state, FC values between frontal and temporal lobes at post-treatment correlated with executive function performance at post-treatment. Additionally, during the learning fMRI task, the brain activation values after treatment correlated with the visual memory performance at post-treatment [38].

These studies suggest that brain activity changes are possible after a cognitive rehabilitation program in PD. Further studies are needed to replicate and complement these findings.

### 3.3. Long-Term Effects of Cognitive Rehabilitation

Furthermore, the ultimate goal of cognitive treatments is to ensure that benefits are maintained over long periods of time, but little is known about the maintenance of cognitive improvements over time in PD patients, and a few studies have evaluated it [39–42, 45]. A summary of these studies is shown in Table 3.

The first study to evaluate the long-term effects of cognitive rehabilitation was published in 2004 by Sinforiani et al. and showed that PD patients attending a cognitive training program combined with motor training during one month showed maintenance of the cognitive benefits after 6 months [39]. However, the study did not include statistical analyses for the follow-up period. Moreover, this study did not include a control group; therefore, we cannot conclude that these possible benefits were related to the cognitive or motor training or the combination of treatments.

Another study in PD compared three training groups: “group A” which attended cognitive training, “group B” which attended cognitive training and transfer training, and “group C” which attended cognitive, transfer, and motor training [40] (Table 3). The authors found that the three groups benefited from training, but those PD patients that attended cognitive training combined with transfer training and physical activity benefited significantly more in the short term. Moreover, over the next 6 months, patients from “group C” were more motivated to spend more time training at home compared to the other groups and showed greater maintenance of cognitive improvements after 6 months [40]. However, because patients from group C spent more hours in training at home over the next 6 months compared to the other groups, these results may be influenced by the difference of hours spent in training. Finally, this study also included an intervention therapy with caregivers focusing on psychoeducation, which helped the patients to continue the training tasks at home [40] (Table 3).

A later study in PD assessed the long-term effects of cognitive rehabilitation for a longer period of time (12 months) [42]. At baseline, these PD patients were randomized to a structured cognitive rehabilitation program (NEUROvitalis), to a nonstructured cognitive rehabilitation program (mentally fit), or to a control group. After 12 months from post-treatment, PD patients that attended NEUROvitalis training program showed reduced cognitive performance compared to post-treatment, but scores were similar compared to baseline. Moreover, the risk of conversion to MCI was found higher in the control group than in any of the cognitive training groups. Regarding depression, the “mentally fit” group was the only group that showed significant reduction in depressive symptoms after training, but these changes were not maintained at follow-up (Table 3). With all, the authors concluded that a structured cognitive treatment could prevent cognitive decline [42].

Regarding the maintenance of neuroimaging changes, to date, only one study has been published assessing the longitudinal effects of cognitive rehabilitation [41]. PD patients attended a 3-month cognitive rehabilitation program and showed increased brain connectivity and activation in the frontal and temporal lobes after treatment. These patients underwent a neuropsychological and neuroimaging assessment after 18 months from post-treatment. The results showed that not only improvements in cognitive performance and functionality were maintained after 18 months but also increased FC was found at follow-up [41]. In addition, PD patients also showed maintenance of the increased brain activation during the memory paradigm at long-term compared to baseline, but the level of activation at long-term was reduced compared to post-treatment. This study showed promising findings regarding the maintenance of brain changes in a neurodegenerative disease; however, the sample size was small, and the control group was not evaluated in the long term. The results need to be replicated.

These few studies suggest the maintenance of cognitive improvements after attending a cognitive rehabilitation program in PD patients (Table 3). However, literature is scarce in this pathology and more research needs to be done, especially including neuroimaging assessment at follow-up.

## 4. Discussion

The studies on the efficacy of cognitive rehabilitation programs in PD suggest that cognitive rehabilitation programs are effective in improving cognition but further research is needed in this field to clarify its efficacy on functional disability and brain activity changes. Also, very little is known about the long-term maintenance of cognitive changes after rehabilitation. There are few cognitive rehabilitation studies in PD which followed the Consolidated Standards of Reporting Trials (CONSORT) guidelines for randomized controlled trials. These make more difficult to find conclusive findings. Future studies should implement these guidelines in order to improve the research quality and validity of findings.

All randomized controlled trials in PD for cognitive rehabilitation programs point to the efficacy in improving cognition. However, most of them highlighted the small sample size as a limitation, which makes it difficult to generalize the findings. Additionally, all of them used different types of cognitive training programs, with different duration and type of exercises. One of the future steps to be taken towards understanding the efficacy of cognitive rehabilitation is identifying the characteristics that make an integrative cognitive rehabilitation program effective against cognitive impairment. A review of cognitive rehabilitation concluded that better results may be obtained in a group-based format compared to an individual format [28]. However, while most of the rehabilitation studies in PD are group-based, this question has not been directly addressed. In addition, a recent meta-analysis compared the efficacy of standardized with tailored (individualized) cognitive interventions, but found that there were insufficient studies for a statistical comparison [29]. Furthermore, other variables are also to be defined, such as the most appropriate number of sessions, their frequency, and the duration of the treatment. Also, the number of cognitive domains trained may also influence the results. Moreover, predictors of the efficacy of cognitive treatments are useful in the disease to adequate cognitive treatment to the patient. Very few studies have evaluated this aspect, and research is needed in the field.

Regarding patients' characteristics, most of the randomized controlled trials in PD have been performed with PD patients at the early Hoehn and Yahr stages of the disease. Future studies should also include PD patients at more advanced stages to evaluate whether cognitive treatments could also benefit these patients. Interestingly, a study protocol was recently published addressing the efficacy of a cognitive rehabilitation in PD patients with dementia, but results are pending [43].

Moreover, transfer effects to clinical aspects have been found in some cognitive rehabilitation studies in PD; however, other studies found no significant changes. The mechanisms that make possible to transfer benefits to clinical variables are unknown. There is an urgent need of studies analyzing this subject. The last goal of cognitive rehabilitation programs is to improve quality of life of patients. Future studies should also include clinical and functionality scales in pre- and post-treatment neuropsychological assessments.

On the other hand, promising findings have been found regarding brain changes after treatment in PD, which support the efficacy of cognitive rehabilitation programs in the disease. Results showing brain connectivity and activation increments after a cognitive treatment of less than 3 months in patients with a neurodegenerative disease are relevant in the field of neurorehabilitation. Future studies should include the MRI acquisition as part of the protocol assessment to evaluate brain changes after treatment and replicate the findings.

All these changes have been analyzed at follow-up, and some studies found maintenance of these improvements. Future randomized controlled trials should include follow-up periods in order to replicate previous findings and assess whether the improvements after training could be maintained over time. It would be also interesting to examine the maintenance of these changes in PD patients with and without booster sessions.

Another aspect to be taken into account during the rehabilitation process of the patient is the role of the caregiver. Some cognitive rehabilitation studies have included an intervention which focused on psychoeducation with the caregivers of the PD patients [40, 44]. The psychoeducation usually addresses aspects of the disease, patients' care management, information about help aids, and the importance of the self-care [40, 44]. These studies found that the caregivers gain self-confidence and felt more confident to take care of the person with the disease.

In conclusion, cognitive rehabilitation programs have demonstrated to be effective in improving cognitive functions and may also improve functional disability and produce brain changes in patients with PD. In addition, to provide a complete or integrative treatment, the combination of cognitive training with other types of trainings or the intervention with the caregivers should be further analyzed. More research should be performed in the field, with a view to reaching generalized conclusions and including cognitive rehabilitation in the standard of care of PD patients.

## Figures and Tables

**Table 1:** Summary of the randomized controlled trials in assessing the efficacy of cognitive rehabilitation programs in PD.

Authors	Sample	H&Y	Cognitive rehabilitation program				Tests^1^	Predictors of greater improvement	MRI (Table 2)	Results	Long-term follow-up (Table 3)	Limitations/risk of bias
			Duration	Paper-pencil—P Computerized—C	Cogn. domains trained	Format						
París et al. [35]	**28 PD** 16 CR 12 ACG	1–3	**12 sessions** 4 weeks 3 times/week 45 min/session	CR—“SmartBrain tool” (P + C) ACG— speech therapy	(i) Attention (ii) WM (iii) Memory (iv) Psychomotor speed (v) EF (vi) Visuospatial ability (vii) Language (viii) Calculation skills (ix) Culture	Group + home	(i) Attention (ii) WM (iii) EF (iv) Processing speed (v) Verbal memory (vi) Visual memory (vii) Visuoconstruction (viii) Visuospatial ability (ix) Verbal fluency (x) *Depression* (xi) *Quality of life* (xii) *Daily living activities*	—	—	**Improvements** (i) Attention/WM (ii) Information processing speed (iii) Visual memory (iv) Visuospatial ability (v) Visuoconstructive ability (vi) Semantic fluency (vii) EF	—	Small sample size

Edwards et al. [34]	**74 PD** 32 CR 42 CG	1–3	**36 sessions** 3 months 3 times/week 1 h/session	CR—“InSight version of SOPT” (C)	(i) Information processing speed	Home	(i) Speed of processing (self-reported) (ii) Perception of cognitive and everyday functioning (self-reported) (iii) *Depression*	<Age at PD diagnosis >Disease duration	—	**Improvements** (i) Speed of processing	—	No ACG Only 1 domain trained Self-reported test for cognition and functionality

Cerasa et al. [33]	**15 PD** 8 CR 7 ACG	1–3	**12 sessions** 6 weeks 2 times/week 1 h/session	CR—“RehaCom” (C) ACG—visuomotor coordination tapping task. In-house software (C)	(i) Attention (ii) Information processing	Group	(i) Attention/processing speed (ii) EF (iii) WM (iv) Spatial memory (v) Verbal memory (vi) Visuospatial orientation (vii) Verbal fluency (viii) *Depression* (ix) *Anxiety* (x) *Quality of life*	—	Yes	**Improvements** (i) Attention/processing speed (ii) WM	—	Small sample size

Zimmermann et al. [37]	**39 PD** 19 CR 20 ACG	2^a^	**12 sessions** 4 weeks 3 times/week	CR—“CogniPlus” (C) ACG—“Nintendo Wii” (C)	(i) Attention (ii) Working memory (iii) EF	Group	(i) Attention (ii) WM (iii) EF (iv) Episodic memory (v) Visuoconstruction	(i) WM score (ii) Flexibility score	—	(i) ACG improved attention compared to CR	—	Small sample size No change in functionality evaluated
Petrelli et al. [32]	**65 PD** 22 CR-NV 22 CR-MT 21 CG	1–3	12 sessions 6 weeks 2 times/week 90 min/session	P “NEUROvitalis”—NV P “mentally fit”—MF	**NV** (i) Attention (ii) Memory (iii) EF **MF** (i) Attention (ii) Memory (iii) Creativity	Group + individual	(i) Attention (ii) Memory (iii) EF (iv) Visuoconstruction (v) *Depression* (vi) *Quality of life*	—	—	**Improvements** **NV versus CG** (i) Working memory (ii) Short-term memory **Improvements** **MF versus CG** (i) *Depression* **Improvements** **NV versus MT** (i) Working memory	12 months	Small sample size No ACG

Peña et al. [31]	**42 PD** 20 PD-CR 22 PD-ACG	1–3	39 sessions 13 weeks 3 times/week 1 h/session	CR—“REHACOP” (P) ACG—occupational activities (P)	(i) Attention (ii) Memory (iii) Language (iv) EF (v) Social cognition (vi) PS	Group	(i) Processing speed (ii) Memory (iii) Executive functions (iv) Social cognition (v) *Functional disability* (vi) *Apathy* (vii) *Depression*	—	Yes	**Improvements** **PD-CR versus PD-ACG** (i) Processing speed (ii) Visual memory (iii) Social cognition (iv) *Functional disability*	18 months	Small sample size

ACG = active control group; CG = control group; CR = cognitive rehabilitation; EF = executive functions; HC = healthy controls; H&Y = Hoehn and Yahr; MRI = magnetic resonance image; PD = Parkinson's disease; WM = working memory. ^1^Tests assessing mood, clinical, and functionality aspects are shown in *italics*. ^a^Median.

**Table 2:** Summary of randomized controlled trials in assessing brain changes related to cognitive rehabilitation programs in PD.

Authors	MRI sample	H&Y	Cognitive rehabilitation program	MRI acquisition/preprocessing/analysis	MRI statistical analysis	MRI results	Correlation with cognitive measures	Limitations/risk of bias
Cerasa et al. [33]	**15 PD** 8 CR 7 ACG	1–3	RehaCom computer program Training: (i) Attention (ii) Information processing	Resting-state fMRI/ICA in FSL/FC analysis	ANOVA (group × time) Region of interests Dorsolateral PFC Ventrolateral PFC ACC Sup + inf parietal left Caudate Cerebellum	Increased functional activity: (i) Left dorsolateral PFC (executive network) (ii) Superior parietal left (attention network)	—	Small sample size Only one type of MRI acquisition

Díez-Cirarda et al. [38] (cognitive results in [31])	**30 PD** 15 CR 15 ACG	1–3	“REHACOP” program Training: (i) Attention (ii) Memory (iii) Language (iv) EF (v) Social cognition	Resting-state fMRI/ROI-to-ROI approach in CONN toolbox	ANOVA (group × time) Paired *t*-test Whole brain	Increased FC between BA9L-BA20L and BA9R-BA20L	Yes	Small sample size and reduced at long-term follow-up Memory fMRI paradigm results at FWE uncorrected
				Memory fMRI paradigm/model-based approach in SPM		Increased brain activation Learning task: left frontal inferior lobe		
						Increased brain activation Memory task: left middle temporal lobe		
				Diffusion weighted/TBSS in FSL		—		
				T1 weighted/VBM in FSL		—		

ACC = anterior cingulate cortex; ACG = active control group; BA = Brodmann area; CR = cognitive rehabilitation; EF = executive functions; FC = functional connectivity; fMRI = functional magnetic resonance imaging; FSL = FMRIB Software Library; H&Y = Hoehn and Yahr; ICA = independent component analysis; MRI = magnetic resonance image; PD = Parkinson's disease; PFC = prefrontal cortex; TBSS = tract-based spatial statistics; VBM = voxel-based morphometry.

**Table 3:** Summary of cognitive rehabilitation studies in PD with longitudinal follow-up evaluation.

Authors	Sample	H&Y	Cognitive rehabilitation program				Tests^1^	MRI (Table 2)	Results (pre- and posttreatment)	Long-term follow-up (T0/T1/T2)	Limitations/risk of bias
			Duration	Paper-pencil—P Computerized—C	Cogn. domains trained	Format					
Nonrandomized/noncontrolled trials											
Sinforiani et al. [39]	**20 PD**	1.5 ± 0.6	**12 sessions** 6 weeks + 12 h MT	C “TNP”	(i) Attention (ii) Abstract reasoning (iii) Visuospatial	Group	(i) MMSE (ii) Digit span (iii) Corsi's test (iv) Babcock's story (v) FAS phonetic (vi) Raven matrix (vii) WCST (viii) Stroop test	—	**Improvements:** (i) Babcock (recall) (ii) FAS phonetic (iii) Raven matrix	**6 months without training: maintenance (no statistical data)** (i) Babcock (recall) (ii) FAS phonetic (iii) Raven matrix	No CG No differentiation of the efficacy of motor or cognitive training No statistical data at follow-up

Reuter et al. [40]	**222 PD** Gr A—CR = 71 Gr B—CR-TT = 75 Gr C—CR-TT-MT = 76 (plus psychoeducation with caregivers)	2–4	**14 sessions** 4 weeks 4 times/week 60 min/session	P + C Group A: cognitive training Group B: cognitive + transfer training Group C: cognitive + transfer + motor training	(i) Attention (ii) Concentration (iii) EF (iv) WM (v) Memory (vi) Processing speed	Individual	(i) ADAS-Cog (ii) SCOPA-Cog (iii) EF-BADS (iv) PS-PASAT (v) *Depression* (vi) *Anxiety* (vii) *PDQ-39*	—	**Improvements in all groups:** (i) ADAS-Cog (ii) BADS (iii) PASAT **Greater improvements in group C:** (i) ADAS-Cog (ii) SCOPA-Cog (iii) BADS (iv) PASAT	**6 months with home training: group C performed more training sessions (T2 > T0)** (i) ADAS-Cog (ii) SCOPA-Cog (iii) BADS (iv) PASAT	No ACG No PDQ-39 scores at baseline Different number of sessions between posttreatment and long-term evaluation

Adamski et al. [45]	**6 PD-CR** **12 HC-CR** **7 HC-CG**	—	**16 sessions** 4 weeks 4 times/week 45 min/session	C “BrainStim”	(i) WM (ii) Encoding (iii) Recall (iv) EF (v) Visuospatial ability	Group	(i) Attention (ii) WM (iii) Short-term memory (iv) Long-term memory (v) PS (vi) EF (vii) *Depression* (viii) *Fatigue*	—	**Improvements in PD group:** (i) Short-term memory (ii) Long-term memory **Improvements in HC groups:** (iii) Diverse cognitive domains	**3 months without training: PD group increased (T2 > T0)** (i) Short-term memory	Small sample size No PD-ACG Baseline differences between groups

Randomized controlled trials											
Petrelli et al. [42]	**65 PD** 22 CR-NV 22 CR-MF 21 CG	1–3	**12 sessions** 6 weeks 2 times/week 90 min/session	P “NEUROvitalis”—NV P “mentally fit”—MF	**NV** (i) Attention (ii) Memory (iii) EF **MF** (i) Attention (ii) Memory (iii) Creativity	Group + individual	(i) Attention (ii) Memory (iii) EF (iv) Visuoconstruction (v) *Depression* (vi) *Quality of life*	—	**Improvements in NV versus CG:** (i) Working memory (ii) Short-term memory **Improvements in MF versus CG:** (i) *Depression* **Improvements in NV versus MT:** (i) Working memory	**12 months without training: NV group maintenance (T2 = T0)** (i) MMSE (ii) DemTect	Small sample size No ACG Long-term evaluation with screening tests

Díez-Cirarda et al. [41]	**42 PD** 20 PD-CR 22 PD-ACG	1–3	**39 sessions** 13 weeks 3 times/week 60 min/session	CR—“REHACOP” (P) ACG—occupational activities (P)	(i) Attention (ii) Memory (iii) Language (iv) EF (v) Social cognition (vi) PS	Group	(i) PS (ii) Memory (iii) Executive functions (iv) Social cognition (v) *Functional disability* (vi) *Apathy* (vii) *Depression*	Yes	**Improvements in PD-CR versus PD-ACG:** (i) PS (ii) Visual memory (iii) Social cognition (iv) *Functional disability* (v) Brain functional changes	**18 months without training: PD-CR increased (T2 > T0):** (i) Verbal memory (ii) Visual memory (iii) EF (iv) ToM (v) *Functional disability* (vi) Brain functional activity	Small sample size Absence of CG at follow-up

ACG = active control group; CG = control group; CR = cognitive rehabilitation; EF = executive functions; FAS = phonetic fluency test; HC = healthy controls; H&Y = Hoehn and Yahr; MMSE = minimental state examination; MRI = magnetic resonance image; MT = motor training; TT = transfer training; PD = Parkinson's disease; PS = processing speed; WCST = Wisconsin card sorting task; WM = working memory. ^1^Tests assessing mood, clinical, and functionality aspects are shown in *italics*.
